# Reliability of Isokinetic Hip Flexor and Extensor Strength Measurements in Healthy Subjects and Athletes: A Systematic Review and Meta-Analysis

**DOI:** 10.3390/ijerph182111326

**Published:** 2021-10-28

**Authors:** Guido Contreras-Díaz, Luis Javier Chirosa-Ríos, Ignacio Chirosa-Ríos, Leonardo Intelangelo, Daniel Jerez-Mayorga, Darío Martinez-Garcia

**Affiliations:** 1 Department Physical Education and Sports, Faculty of Sport Science, University of Granada, 18011 Granada, Spain; guido.contreras@ulagos.cl (G.C.-D.); lchirosa@ugr.es (L.J.C.-R.); ichirosa@ugr.es (I.C.-R.); 2 Department of Health, Los Lagos University, Puerto Montt 5500000, Chile; 3 Musculoskeletal Research Group, University Center for Assistance, Teaching and Research, University of Gran Rosario, Rosario S2000, Argentina; leonardo.intelangelo@gmail.com; 4 Exercise and Rehabilitation Sciences Laboratory, School of Physical Therapy, Faculty of Rehabilitation Sciences, Universidad Andres Bello, Santiago 7591538, Chile; damaga1991@gmail.com

**Keywords:** reliability, reproducibility, hip, isokinetic, muscle strength

## Abstract

Background: The objective of this systematic review and meta-analysis was to examine the reliability of isokinetic measurements of hip strength in flexion and extension in healthy subjects and athletes. Methods: The databases used were Web of Science, SCOPUS, Medline and PubMed. R was used for all statistical analyses. Results: Hip flexion shows moderate reliability in the supine position (ICC = 0.72; 95% CI: 0.46–0.99) and good reliability in the standing position (ICC = 0.79; 95% CI: 0.54–1.04). Hip extension shows excellent reliability in the supine position (ICC = 0.90; 95% CI: 0.85–0.96) and moderate reliability in the standing position (ICC = 0.72; 95% CI: 0.48–0.96). Flexion of 120°/s and 180°/s showed excellent reliability (ICC = 0.93; 95% CI: 0.85–1.00), (ICC = 0.96; 95% CI: 0.92–1.01). The 60°/s and 120°/s extension showed good reliability (ICC = 0.90; 95% CI: 0.82–0.98), (ICC = 0.87; 95% CI: 0.75–0.99). The 180°/s extension presented excellent reliability (ICC = 0.93; 95% CI: 0.82–1.03). Conclusions: The standing position shows good reliability for hip flexion and the supine position shows excellent reliability for hip extension, both movements have excellent reliability at velocities between 120°/s to 180°/s.

## 1. Introduction

Optimal muscle strength levels are directly related to sports performance and rehabilitation [1]. In terms of sports performance, it has been shown that runners with greater hip extensor strength have greater anterior trunk inclination when running, which improves running mechanics, reduces the work done by the knee extensors and consequently decreases the probability of knee injury [2]. Likewise, these muscles have a great participation during propulsion in jumping, contributing 31.2% of the work in vertical jumping and 44.2% of the work in horizontal jumping [3]. On the other hand, having greater cross-sectional area (CSA) in the hip flexor muscles is related to greater performance during sprinting in pre-adolescent [4] and adult male [5] sprinters, so having strong hip flexor muscles increases running speed [6]. In the hip, it has been shown that strength deficit is associated with lower extremity injuries [7]. When the deficit is in hip flexors (HF), it is manifested through pathologies such as femoroacetabular impingement [8] and anterior cruciate ligament injuries [9]. When the deficit is in hip extensor (HE), it is manifested through pathologies such as patellofemoral dysfunction [10] and Achilles tendinopathy [11].

For muscle strength assessment, isokinetic dynamometers are the gold standard [12], they allow us to establish agonist/antagonist ratios through different angular velocities and are also considered an effective means to rehabilitate and condition muscle function [13]. Isokinetic dynamometry arrived in the late 1960s with the first Cybex I and since then, a great deal of research has been generated in the field of rehabilitation and sports performance [14] with the knee joint being the most studied and to a lesser extent the hip [15].

Additionally, as isokinetic dynamometry is widely used, it should be noted that its usefulness depends on how reproducible its measurements are [16]. It is known that isokinetic measurements are reproducible in the shoulder [17], knee [18] and spine joints [19]; however, studies on the hip joint are scarce and sometimes contradictory [20]. Therefore, it is important to know the reliability of isokinetic evaluations at the hip level that allow us to determine the strength levels in this joint.

The reliability of hip flexion and extension force measurement with an isokinetic dynamometer has not been thoroughly investigated; therefore, the objective of this systematic review was to (I) examine the reliability of isokinetic flexion and extension force measurements in healthy subjects and athletes; (II) determine which position is the most valid and reliable for force measurement; and (III) select the most reliable velocity for assessing hip flexor and extensor strength.

## 2. Methods

### 2.1. Study Design

**Figure 1:**
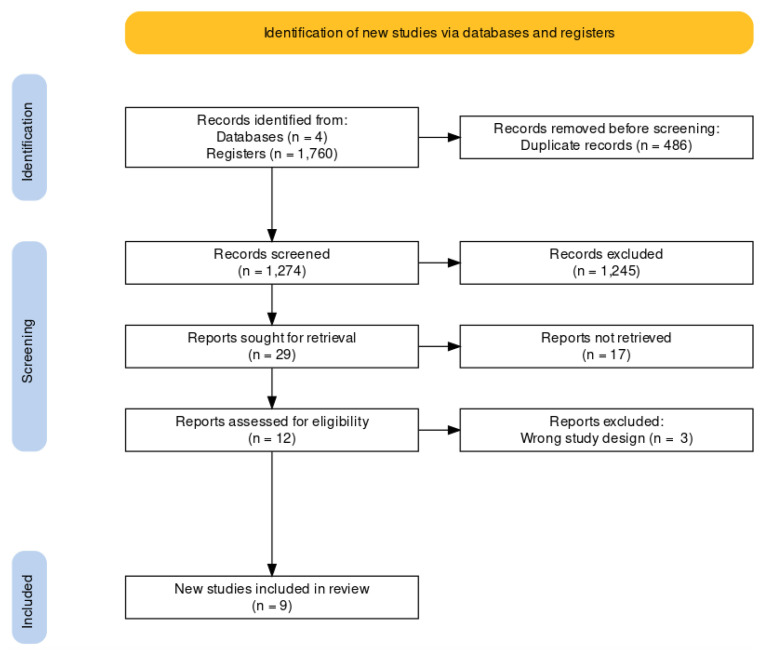
PRISMA flow diagram.

A systematic revision and meta-analysis were carried out on 14 July 2021 to summarize the current knowledge regarding test–retest reliability (as measured by the ICC) of isokinetic hip strength testing in physically active and/or athletic adults and adolescents. We included quantitative and qualitative summaries: (1) a quantitative meta-analysis to estimate the reliability of present tests for hip flexion-extension and (2) a qualitative review of factors influencing reliability. Before starting the review, a protocol was registered in the International Prospective Register of Systematic Reviews (PROSPERO) registration number CRD42020199520. This systematic review’s reporting flow diagram was based on the Preferred Reporting Items for Systematic reviews and Meta-Analyses (PRISMA) guidelines [21] (Figure 1).

### 2.2. Search Strategy

Original quantitative research studies were identified through searching the five principal electronic databases: Web of Science, SCOPUS, MedLine and PubMed. The bibliographic search was carried out by combining the different Medical Subject Headings (MeSH) terms with the following keywords: “Isokinetic”, “Dynamometer”, “Hip”, “Reliability” and “Reproducibility”. These search terms were combined with two Boolean operators AND, OR. The bibliographies of other previous related reviews and the studies finally selected were examined to search for new studies. Other possible scientific evidence related to the subject was identified by contacting authors of the published articles through email.

Two authors examined the articles’ title/summary found in the databases. After the initial selection, they analyzed each study with the inclusion criteria. Each criterion was evaluated as yes/no. If discrepancies existed between the authors, the articles’ ratings were shared and discussed until a consensus was reached. The authors were familiar with the existing literature and did not have a different bias with any of the studies selected for inclusion in the review.

### 2.3. Eligibility Criteria

Original quantitative research was eligible for inclusion in the quantitative meta-analysis if (1) studies were in English or Spanish language; (2) the subjects were healthy, physically active adults and athletes; (3) isokinetic test of hip flexion/extension were evaluated; (4) mean ICC values, as well as a number of subjects and test (used for estimating variance), could be readily determined from the text. The articles that met the inclusion criteria were identified and their full-text versions were obtained. For studies where multiple ICC results were presented, a typical value was sought for the quantitative analysis (i.e., 10–15 min duration, inter-session interval >1 day and <1 month, median result from multiple pipelines).

### 2.4. Evaluation of the Methodological Quality of the Studies Included

The methodological quality of the selected studies was evaluated using a critical appraisal tool (CAT) [22] and through the quality assessment of reliability studies (QAREL) [23].

The CAT scale contains items of validity and reliability to evaluate the methodological quality of the studies. There are 13 evaluation points, of which four points refer to validity and nine points to the reliability, the latter being used for the review. A column was added that evaluated each study’s final result in percentage (%), a maximum rating of 90% is considered the highest methodological quality and a score over 45% is considered a high-quality study [22].

The QAREL scale contains 11 points. Points 1 and 2 consider the bias of the sample and the representativeness of the subjects and qualifiers, points 3 to 7 correspond to the blinding of the qualifiers, point 8 refers to the order in which the subjects were evaluated, point 9 considers the time interval in which the subjects were evaluated, point 10 evaluates whether the test was applied and interpreted adequately and point 11 refers to the statistical analysis [23]. A column was added that evaluated each study’s final result as a percentage (%), with a maximum of 110% considered the highest methodological quality.

### 2.5. Data Collection Process

R was used for all statistical analyses [24]. Excel data was extracted with the read.xls function in gdata [25]. The metafor package was used to perform the analysis meta-analysis results [26]. The rma.mv function was used to compute a meta-analytic estimate of the population ICC with studies nested by authors; random effects were specified for the dataset and the resulting model was fit using restricted maximum likelihood estimation. This procedure has been documented by Noble et al. [27] for formal meta-analysis of ICC values. Therefore, two assumptions were made to conduct an ICC-based meta-analysis. First, a meta-analysis was performed using the raw ICC values with the assumption that these were distributed normally. While not exact, this assumption is often made in the similar case of meta-analysis with Pearson’s correlation coefficient and tends to be less skewed when values are far from one. Second, we assumed that each study’s ICC variance could be approximated as Donner, 1986; via Shoukri et al. [28] established.

Forest plots of all studies included in the meta-analysis were created with the forest function. A funnel plot showing the relationship between ICC coefficients and their estimated standard errors was created with the funnel function. Heterogeneity was assessed with Cochrane’s Q and publication bias was assessed by estimating funnel plot asymmetry via the ranked regression test (rank test function).

The researchers’ data extraction included: number of subjects, gender, type of subject, unilateral or bilateral hip evaluation, and the time between the re-test and the dynamometer used (Table 1).

## 3. Results

### 3.1. Study Selection

A total of 1760 studies were identified through an electronic database search (PubMed, n = 146, Web of Science, n = 286, Medline, n = 145, Scopus, n = 189), of which 486 duplicate articles were identified and eliminated. After reading the title and the abstract, 1245 articles were eliminated, leaving 29 studies for full reading, but 17 were eliminated for not meeting inclusion criteria, leaving 12 articles, of which three were eliminated for not having a test-retest. Therefore, a total of nine studies on hip isokinetic assessment were included in this systematic review (Figure 1).

### 3.2. Characteristics of the Studies

From each manuscript selected for review, the following information was considered: number of subjects, gender, type of subjects (healthy and/or athletic), unilateral or bilateral hip assessment, the time between test–retest and the dynamometer used during the assessment (Table 1). The sample size of the selected studies was between 10 and 52 subjects, aged between 6 and 45 years, all healthy and/or physically active. Selected studies used the following isokinetic dynamometers; Cybex II [29], Biodex Medical System [30,31], biodex system 3 [32], Cybex 340 [33], Cybex Norm [34], CON-TREX [35], biodex [36] and Biodex System 3 Pro [37]. The reliability data extracted included: author, year, movement, position, velocity (°/s), intraclass correlation coefficient (ICC, 95%CI), standard error of measurement (SEM, Nm) for concentric flexion (Table 2), concentric extension (Table 3) and eccentric flexion/extension (Table 4).

### 3.3. Risk of Bias in Studies

The quality of studies evaluated through the CAT scale obtained a score between 56% and 78%, of which eight articles had a high-quality evaluation (Table 5). The quality of the studies evaluated through QAREL obtained a score between 40% and 60% (Table 6).

### 3.4. Anatomical Plane and Motion

Seven studies [29,30,32,33,34,36,37] assessed hip strength using two planes: sagittal plane (flexion/extension movements) and frontal plane (abduction/adduction movements). Two studies [31,35] evaluated hip strength only in the sagittal plane, performing flexion and extension.

### 3.5. Muscle Contraction

Three studies [30,33,35] presented concentric and eccentric strength measurements for the hip, two studies [31,36] evaluated concentrically and isometrically and four studies [29,32,34,37] only evaluated in concentric mode.

### 3.6. Position

Hip flexion and extension were evaluated in two different positions: supine and standing. The most used position was supine [29,31,33,34,35,36] and, to a lesser extent, the bipedal position [30,32,37].

### 3.7. Velocity

The velocity used for the concentric phase was between 30°/s and 180°/s and the velocity used for the eccentric phase was between 30°/s and 90°/s. For the concentric phase, two studies [29,32] used a speed of 30°/s, six studies [30,33,34,35,36,37] used a speed of 60°/s, one study [29] used a speed of 90°/s, three studies [31,33,36] used a speed of 120°/s, one study [32] used a speed of 150°/s and two studies [35,37] used a speed of 180°/s. For the eccentric phase, two studies [30,33] used a speed of 60°/s and one study [35] used a speed of 30 and 90°/s.

### 3.8. Reliability

For this review, we suggest that ICC values below 0.5 indicate low reliability, values between 0.5 and 0.75 indicate moderate reliability, values between 0.75 and 0.9 indicate good reliability and values above 0.90 indicate excellent reliability [38].

**Figure 2:**
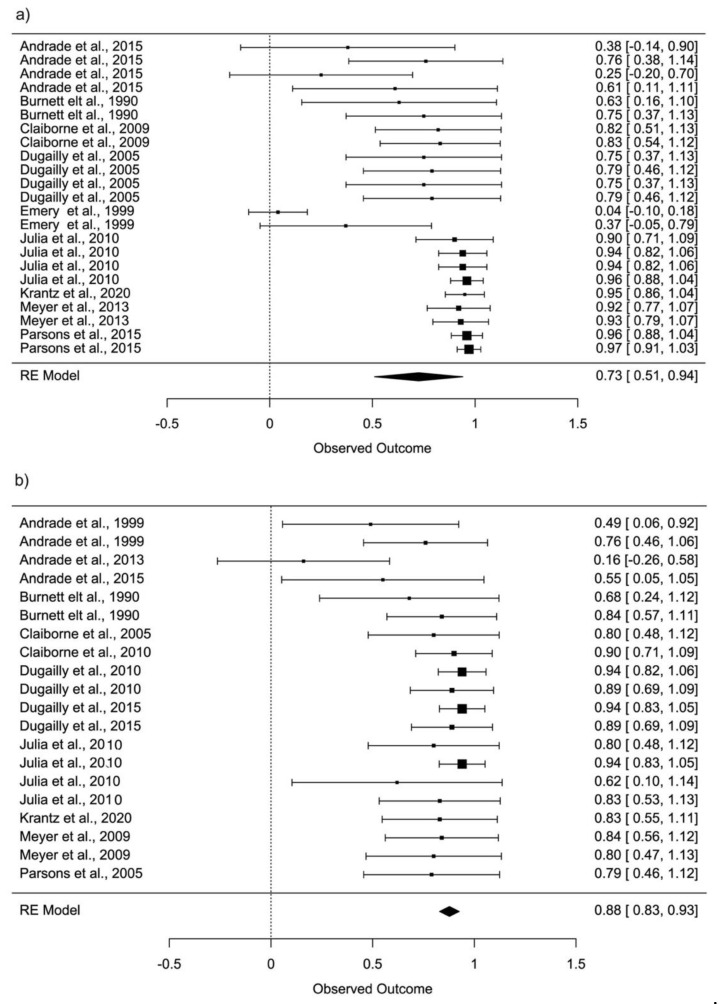
Forest plot, 95% confidence interval (CI95%) for reliability hip flexion (**a**) and reliability hip extension (**b**).

When all selected studies were combined, the estimated mean reliability of hip flexion was found to be moderate (ICC = 0.73; 95% CI: 0.51–0.94), in contrast to hip extension which was found to be good (ICC = 0.88; 95% CI: 0.83–0.93) (Figure 2).

**Figure 3:**
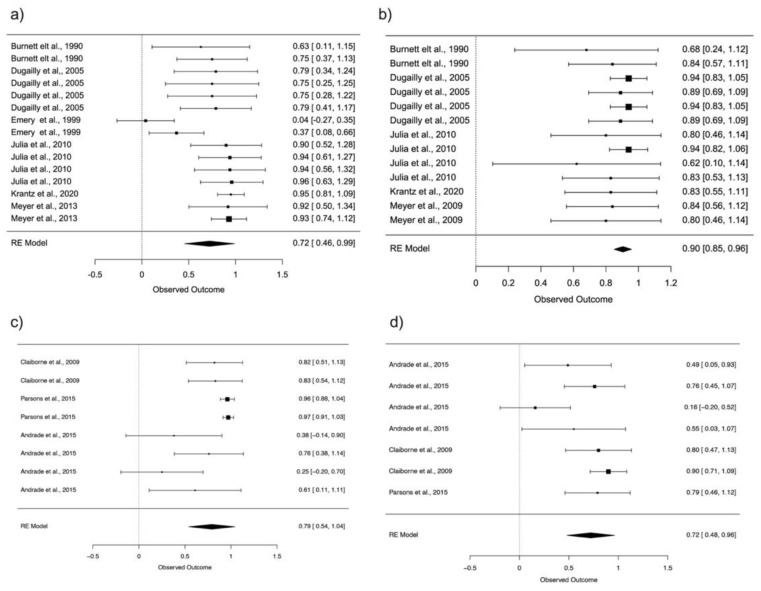
Forest plot, 95% confidence interval (CI95%) for reliability hip flexion in supine position (**a**), reliability hip extension in supine position (**b**), reliability hip flexion in standing position (**c**) and reliability hip extension in standing position (**d**).

In case other variables are considered that may cause a bias, such as the position of the subject during the tests, we note that the two most used positions for hip strength assessment are supine and standing. Hip flexion shows moderate reliability in the supine position (ICC = 0.72; 95% CI: 0.46–0.99) and good reliability in the standing position (ICC = 0.79; 95% CI: 0.54–1.04). Hip extension shows excellent reliability in the supine position (ICC = 0.90; 95% CI: 0.85–0.96) and moderate reliability in the standing position (ICC = 0.72; 95% CI: 0.48–0.96) (Figure 3).

As mentioned above, another of the variables considered most relevant to the reliability of the tests is the speed of the repetitions performed. There is a wide variety of velocities used for hip strength assessment. These range from 30°/s to 180°/s, with multiple others in between. To understand a little about how this variable affects the variety, we can see that the most reliable speeds are intermediate and high speeds.

**Figure 4:**
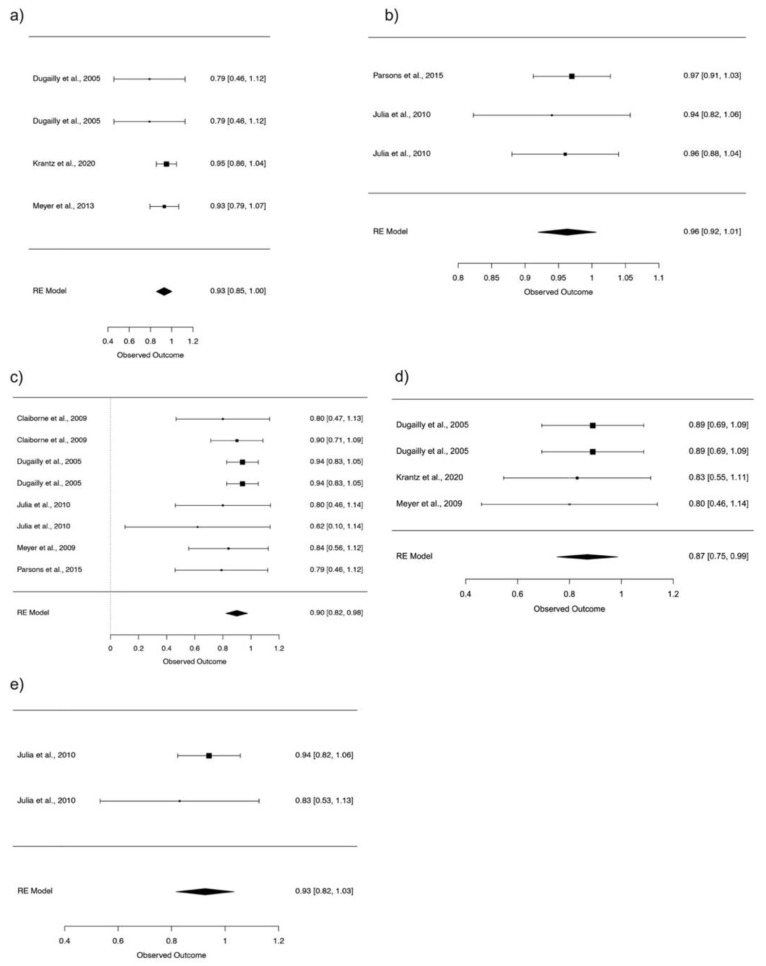
Forest plot, 95% confidence interval (CI95%) for reliability hip flexion 120°/s (**a**), reliability hip flexion 180°/s (**b**), reliability hip extension 60°/s (**c**) reliability hip extension 120°/s (**d**) and reliability hip extension 180°/s (**e**).

The 120°/s and 180°/s flexion showed excellent reliability (ICC = 0.93; 95% CI: 0.85–1.00), (ICC = 0.96; 95% CI: 0.92–1.01). The 60°/s and 120°/s extension presented good reliability (ICC = 0.90; 95% CI: 0.82–0.98), (ICC = 0.87; 95% CI: 0.75–0.99). The 180°/s extension presented excellent reliability (ICC = 0.93; 95% CI: 0.82–1.03) (Figure 4).

## 4. Discussion

This systematic review aimed to (I) examine the reliability of isokinetic flexion and extension strength measurements in healthy subjects and athletes; (II) determine which position is the most valid and reliable for strength measurement; and (III) select the most reliable velocity for assessing hip flexor and extensor strength. The main finding of the present study revealed (I) The reliability of isokinetic strength measurement is moderate in flexion (ICC = 0.73) and good in extension (ICC = 0.88); (II) Standing position presents good reliability in hip flexion (ICC = 0.79) and moderate reliability in hip extension (ICC = 0.72); (III) Supine position presents moderate reliability in hip flexion (ICC = 0.72) and excellent reliability in hip extension (ICC = 0.90); (IV) in flexion the velocity of 120°/s and 180°/s have excellent reliability (ICC = 0.93), (ICC = 0.96) and (V) in extension the velocity of 180°/s show excellent reliability (ICC = 0.93). Isokinetic evaluations of hip flexion and extension movements have moderate to good reliability depending on the position and velocity at which they are performed. 

### 4.1. Movement and Position

Hip flexion and extension movements performed in the sagittal plane using an isokinetic device can be performed in two positions: supine position and standing position, with the supine position being the most frequently used [29,31,33,34,35,36] and the one with the highest reliability during hip extension movement (ICC = 0.90) according to the results of the meta-analysis. Other authors, such as Abdelmohsen et al. [39] also used the supine position to compare the isokinetic strength of the hip flexor and extensor muscles of the dominant side versus the non-dominant side, finding no significant differences between the two sides. Sugiura et al. [40] measured hip extensor, knee extensor and knee flexor strength in elite sprinters to establish a relationship between strength deficits and hamstring injury by measuring hip extension in a standing position, simulating the sprinting motion, finding an association between hamstring injury and the ratio of eccentric hamstring strength to concentric hip extensor strength. Ambegaonkar et al. [41] studied the relationship between core endurance, hip strength and balance in female college athletes using a hand-held dynamometer to measure hip strength in the three planes of motion, evaluating seated hip flexion and bipedal hip extension, based on the positions recommended by manual muscle testing [42], finding that bilateral hip flexion and extension were positively correlated with anterior balance scores (anterior SEBT).

### 4.2. Velocity

The velocity used for the concentric phase was between 30°/s and 180°/s and for the eccentric phase was between 30°/s and 90°/s, with the most used velocity being 60°/s for both phases [30,33,34,35,36,37]. The results of the meta-analysis show that for hip flexion, the velocities with the highest reliability are 120°/s (ICC = 0.93) and 180°/s (ICC = 0.96) while for hip extension, the velocities with the highest reliability correspond to 60°/s (ICC = 0.90), 120°/s (ICC = 0.87) and 180°/s (ICC = 0.93). Other authors, such as Calmels et al. [43] studied the relationship between flexion/extension torque in hip, knee and ankle of healthy subjects, using concentric velocity of 60°/s, 120°/s and 240°/s and eccentric velocity of 60°/s and 120°/s for all joints, finding no significant differences between the left/right side flexion-extension torque ratios for hip and knee at all angular velocities in concentric and eccentric mode. Arokoski et al. [44] studied muscle strength and the cross-sectional area in men with and without hip osteoarthritis, evaluated abduction, adduction, flexion and extension isometrically and isokinetically (60°/s and 120°/s) in supine position, finding good reliability for flexion at 60°/s (ICC = 0. 70) and 120°/s (ICC = 0.89), excellent reliability for extension at 60°/s (ICC 0.90) and good reliability for extension at 120°/s (0.84) in healthy subjects. Subjects with osteoarthritis also had good reliability for flexion at 60°/s (ICC = 0.84), 120°/s (ICC = 0.89) and extension at 60°/s (ICC = 0.87) and 120°/s (ICC = 0.86). Eng et al. [45], in their reliability study of lower extremity strength measurements in people with chronic stroke, measured isokinetic hip, knee and ankle flexion and extension strength of the hemiparetic side and the healthy side at 60°/s in semi-reclined position, finding high ICCs for peak torque and average torque (0. 95–0.98 for peak torque and 0.88–0.96 for average torque) in hip, knee and ankle flexion and extension movements on the healthy side and high ICC for the hemiparetic side (0.97–0.99 for peak torque and 0.96–0.98 for average torque) during the same movements.

The limitations of these studies have to do with the variability of the population, ranging from children to adults, trained and untrained subjects, with and without experience in the evaluation, which influences the overall or total reliability of the meta-analysis. However, despite the heterogeneity and methodological quality of the studies, we were able to identify positions and velocities with good and excellent reliability, which guides the health and sport professional to make decisions and standardize processes during the evaluation.

## 5. Conclusions

The reliability of isokinetic hip assessments is determined by factors such as subject position, speed of movement, muscle contraction and pelvic stability [29,30,31,32,33,34,35,36,37]; however, according to the results of the meta-analysis there are measurements with higher reliability:

- Hip flexion shows good reliability in the standing position (ICC = 0.79; 95% CI: 0.54–1.04).
- Hip extension shows excellent reliability in supine position (ICC = 0.90; 95% CI: 0.85–0.96).
- Hip flexion at 120°/s and 180°/s shows excellent reliability (ICC = 0.93; 95% CI: 0.85–1.00), (ICC = 0.96; 95% CI: 0.92–1.01).
- Hip extension at 60°/s and 120°/s show good reliability (ICC = 0.90; 95% CI: 0.82–0.98), (ICC = 0.87; 95% CI: 0.75–0.99) and excellent reliability at 180°/sond (ICC = 0.93; 95% CI: 0.82–1.03).

Despite these results, it is necessary to carry out more studies with higher methodological quality in different populations, of different ages and sexes, with different modalities of muscle contraction and in both extremities, which will allow us to confirm our results and reproduce them systematically.

## Figures and Tables

**Table 1:** Characteristics of the participant.

Study	N	Gender	Type Subjects	Bilateral	Time Rest	Dynamometer
Burnett et al. [29]	29	Boys	Healthy	No	7–14 days	Cybex II
Claiborne et al. [30]	13	Boys/Girls	Healthy	Yes	7 days	Biodex Medical System
Krantz et al. [31]	30	Men/Woman	Healthy	Yes	7 days	Biodex Medical System
Dos Santos Andrade et al. [32]	17	Woman	Soccer Players	Yes	7 days	Biodex System 3
Dugailly et al. [33]	28	Boys/Girls	Sports-active	Yes	10 min	Cybex 340
Emery et al. [34]	19	Males	Healthy	Yes	7 days	Cybex Norm
Julia et al. [35]	10	Males/Woman	Healthy	Yes	7 days	CON-TREX
Meyer et al. [36]	10	Males/Woman	Healthy	No	7 days	Biodex
Parsons et al. [37]	52	Boys/Girls	Healthy	No	7 days	Biodex System 3 Pro

**Table 2:** Reliability and absolute reliability of concentric flexion hip.

Study	Hip Action Evaluated	Posture	Speed (°/s)	Mean/(SD) 1° Test	Mean/(SD) 2° Test	Mean/(SD) 3° Test	ICC (95% CI)	Limits ICC	SEM (Nm)	SEM (%)	SRD (Nm)	SRD (%)
Burnett et al. [29]	Flexion	Supine	30	6.2	6.0	(-)	0.63	(-)	(-)	(-)	(-)	(-)
	Flexion	Supine	90	5.1	5.2	(-)	0.75	(-)	(-)	(-)	(-)	(-)
Claiborne et al. [30]	Flexion Left	Biped	60	31.37	34.05	(-)	0.82	0.80–0.90	13.92	(-)	(-)	(-)
	Flexion Right	Biped	60	37.49	25.89	(-)	0.83	0.80–0.90	13.16	(-)	(-)	(-)
Dos Santos Andrade et al. [32]	Flexion Right	Biped	30	218.9	208.5	(-)	0.25	(-) 0.26–0.65	(-)	(-)	(-)	(-)
	Flexion Left	Biped	30	225.3	217.4	(-)	0.38	(-) 0.13–0.73	(-)	(-)	(-)	(-)
	Flexion Right	Biped	150	208.7	203.2	(-)	0.61	0.18–0.84	(-)	(-)	(-)	(-)
	Flexion Left	Biped	150	197.7	190.5	(-)	0.76	0.44–0.91	(-)	(-)	(-)	(-)
Dugailly et al. [33]	Flexion Women	Supine	60	(-)	(-)	(-)	0.75	(-)	3.7	(-)	(-)	(-)
	Flexion Women	Supine	120	(-)	(-)	(-)	0.79	(-)	3.6	(-)	(-)	(-)
	Flexion Men	Supine	60	(-)	(-)	(-)	0.75	(-)	3.7	(-)	(-)	(-)
	Flexion Men	Supine	120	(-)	(-)	(-)	0.79	(-)	3.6	(-)	(-)	(-)
Emery et al. [34]	Flexion Right	Supine	60	54.2	62.9	51.0	0.37	0.06–0.64	(-)	(-)	(-)	(-)
	Flexion Left	Supine	60	52.4	58.3	43.1	0.04	0–0.35	(-)	(-)	(-)	(-)
Julia et al. [35]	Flexion Left	Supine	60	(-)	(-)	(-)	0.90	(-)	(-)	(-)	(-)	(-)
	Flexion Left	Supine	180	(-)	(-)	(-)	0.94	(-)	(-)	(-)	(-)	(-)
	Flexion Right	Supine	60	(-)	(-)	(-)	0.94	(-)	(-)	(-)	(-)	(-)
	Flexion Right	Supine	180	(-)	(-)	(-)	0.96	(-)	(-)	(-)	(-)	(-)
Krantz et al. [31]	Flexion	Supine	120	28.29	28.67	(-)	0.83	0.89–0.97	1.76	6.2	(-)	(-)
Meyer et al. [36]	Flexion	Supine	60	120.7	124.7	(-)	0.92	0.80–0.97	10.51	8.57	29.13	23.75
	Flexion	Supine	120	103.3	107.1	(-)	0.93	0.82–0.97	8.41	8.00	23.32	22.17
Parsons et al. [37]	Flexion	Biped	60	74.8 (28.5)	70.1 (25.4)	(-)	0.96	0.91–0.98	7.1	(-)	(-)	(-)
	Flexion	Biped	180	194.1 (74.6)	189.0 (72.6)	(-)	0.97	0.95–0.99	16.7	(-)	(-)	(-)

ICC = intraclass correlation coefficient (95% confidence interval); SEM = standard error of measurement; SRD = smallest real difference; (-) = not available.

**Table 3:** Relative and absolute reliability of concentric extension hip.

Study	Hip Action Evaluated	Posture	Speed (°/s)	Mean/(SD) 1° Test	Mean/(SD) 2° Test	Mean/(SD) 3° Test	ICC (95% CI)	Limits ICC	SEM (Nm)	SEM (%)	SRD (Nm)	SRD (%)
Burnett et al. [29]	Extension	Supine	30	10.3	12	(-)	0.68	(-)	(-)	(-)	(-)	(-)
	Extension	Supine	90	10.1	12.3	(-)	0.84	(-)	(-)	(-)	(-)	(-)
Claiborne et al. [30]	Extension Left	Biped	60	32.22	34.55	(-)	0.80	0.80–0.90	14.84	(-)	(-)	(-)
	Extension Right	Biped	60	33.78	31.77	(-)	0.90	0.80–0.90	10.40	(-)	(-)	(-)
Dos Santos Andrade et al. [32]	Extension Right	Biped	30	239.0	227.4	(-)	0.16	(-) 0.35–0.59	(-)	(-)	(-)	(-)
	Extension Left	Biped	30	258.8	250.4	(-)	0.49	0.01–0.79	(-)	(-)	(-)	(-)
	Extension Right	Biped	150	228.8	227.7	(-)	0.55	0.09–0.82	(-)	(-)	(-)	(-)
	Extension Left	Biped	150	237.5	235.4	(-)	0.76	0.44–0.91	(-)	(-)	(-)	(-)
Dugailly et al. [33]	Extension Women	Supine	60	(-)	(-)	(-)	0.94	(-)	9.5	(-)	(-)	(-)
	Extension Women	Supine	120	(-)	(-)	(-)	0.89	(-)	8.2	(-)	(-)	(-)
	Extension Men	Supine	60	(-)	(-)	(-)	0.94	(-)	9.5	(-)	(-)	(-)
	Extension Men	Supine	120	(-)	(-)	(-)	0.89	(-)	8.2	(-)	(-)	(-)
Julia et al. [35]	Extension Left	Supine	60	(-)	(-)	(-)	0.80	(-)	(-)	(-)	(-)	(-)
	Extension Right	Supine	60	(-)	(-)	(-)	0.62	(-)	(-)	(-)	(-)	(-)
	Extension Left	Supine	180	(-)	(-)	(-)	0.94	(-)	(-)	(-)	(-)	(-)
	Extension Right	Supine	180	(-)	(-)	(-)	0.83	(-)	(-)	(-)	(-)	(-)
Krantz et al. [31]	Extension	Supine	120	51.54	54.74	(-)	0.83	0.67–0.92	7.22	13.6	(-)	(-)
Meyer et al. [36]	Extension	Supine	60	120.2	140.7	(-)	0.84	0.61–0.93	12.66	9.70	35.10	26.90
	Extension	Supine	120	114.9	132.1	(-)	0.80	0.55–0.92	16.11	13.06	44.65	36.31
Parsons et al. [37]	Extension	Biped	60	52.2 (25.2)	50.6 (25.3)	(-)	0.79	0.63–0.88	15.1	(-)	(-)	(-)

ICC = intraclass correlation coefficient (95% confidence interval); SEM = standard error of measurement; SRD = smallest real difference; (-) = not available.

**Table 4:** Relative and absolute reliability of eccentric flexion and extension hip.

Study	Hip Action Evaluated	Posture	Speed (°/s)	Mean/(SD) 1° Test	Mean/(SD) 2° Test	Mean/(SD) 3° Test	ICC (95% CI)	Limits ICC	SEM (Nm)	SEM (%)	SRD (Nm)	SRD (%)
Flexion Eccentric												
Claiborne et al. [30]	Flexion Left	Biped	60	35.38	34.89	(-)	0.74	0.62–0.79	18.06	(-)	(-)	(-)
	Flexion Right	Biped	60	28.34	34.90	(-)	0.91	0.80–0.91	9.42	(-)	(-)	(-)
Dugailly et al. [33]	Flexion Right	Supine	60	79.5	68.3	60.5	0.28	0.09–0.66	(-)	(-)	(-)	(-)
	Flexion Left	Supine	60	63.2	73.6	53.3	0.35	0.04–0.62	(-)	(-)	(-)	(-)
Extension Eccentric												
Claiborne et al. [30]	Extension Left	Biped	60	30.91	35.44	(-)	0.80	0.80–0.91	14.68	(-)	(-)	(-)
	Extension Right	Biped	60	45.50	34.26	(-)	0.76	0.62–0.79	19.49	(-)	(-)	(-)
Julia et al. [35]	Extension Left	Supine	30	(-)	(-)	(-)	0.68	(-)	(-)	(-)	(-)	(-)
	Extension Right	Supine	30	(-)	(-)	(-)	0.80	(-)	(-)	(-)	(-)	(-)
	Extension Left	Supine	90	(-)	(-)	(-)	0.75	(-)	(-)	(-)	(-)	(-)
	Extension Right	Supine	90	(-)	(-)	(-)	0.78	(-)	(-)	(-)	(-)	(-)

ICC = intraclass correlation coefficient (95% Confidence Interval); SEM = standard error of measurement; SRD = smallest real difference; (-) = Not available.

**Table 5:** Evaluation of the quality of studies with the critical evaluation tool (CAT).

Study	1	2	3	4	5	6	7	8	9	%
Burnett et al. [29]	Y	N	N	N	N	Y	Y	Y	Y	56
Claiborne et al. [30]	Y	N	N	N	N	Y	Y	Y	Y	56
Dos Santos Andrade et al. [32]	Y	Y	N	N	N	Y	Y	Y	Y	67
Dugailly et al. [33]	Y	N	N	N	N	Y	Y	Y	Y	56
Emery et al. [34]	Y	N	N	N	N	Y	Y	Y	Y	56
Julia et al. [35]	Y	N	N	N	N	Y	Y	Y	Y	56
Krantz et al. [31]	Y	Y	N	N	N	Y	Y	Y	Y	67
Meyer et al. [36]	Y	N	N	N	Y	Y	Y	Y	Y	67
Parsons et al. [37]	Y	N	Y	Y	N	Y	Y	Y	Y	78

Y = Yes; N = No. 1. If human subjects were used, did the authors give a detailed description of the sample of subjects used to perform the test? 2. Did the authors clarify the qualification, or competence of the rater(s) who performed the test? 3. If interrater reliability was tested, were raters blinded to the findings of other raters? 4. If intra-rater reliability was tested, were raters blinded to their own prior findings of the test under evaluation? 5. Was the order of examination varied? 6. Was the stability (or theoretical stability) of the variable being measured taken into account when determining the suitability of the time interval between repeated measures? 7. Was the execution of the test described in sufficient detail to permit replication of the test? 8. Were withdrawals from the study explained? 9. Were the statistical methods appropriate for the purpose of the study? %: final percentage of reliability (Items “yes” × 100)/9.

**Table 6:** Evaluation of the quality of studies with the QAREL scale.

Study	P1	P2	P3	P4	P5	P6	P7	P8	P9	P10	P11	%
Burnett et al. [29]	Y	Y	Y	UC	NA	UC	UC	UC	Y	Y	Y	60
Claiborne et al. [30]	Y	Y	UC	UC	NA	UC	UC	N	Y	Y	Y	50
Dos Santos Andrade et al. [32]	Y	Y	UC	UC	NA	UC	UC	N	Y	Y	Y	50
Dugailly et al. [33]	Y	Y	UC	UC	NA	UC	UC	UC	UC	Y	Y	40
Emery et al. [34]	Y	Y	UC	UC	NA	UC	UC	UC	Y	Y	Y	50
Julia et al. [35]	Y	Y	N	UC	NA	UC	UC	N	Y	Y	Y	50
Krantz et al. [31]	Y	Y	N	N	NA	UC	UC	N	Y	Y	Y	50
Meyer et al. [36]	Y	Y	UC	UC	NA	UC	UC	N	Y	Y	Y	50
Parsons et al. [37]	Y	Y	N	Y	NA	UC	UC	N	Y	Y	Y	60

P = Question on the QAREL scale; Y = Yes, complies; N = No, does not comply; UC = Unclear; NA = Not applicable. 1. Was the test evaluated in a sample of subjects who were representative of those to whom the authors intended the results to be applied? 2. Was the test performed by raters who were representative of those to whom the authors intended the results to be applied? 3. Were raters blinded to the findings of other raters during the study? 4. Were raters blinded to their own prior findings of the test under evaluation? 5. Were raters blinded to the results of the reference standard for the target disorder (or variable) being evaluated? 6. Were raters blinded to clinical information that was not intended to be provided as part of the testing procedure or study design? 7. Were raters blinded to additional cues that were not part of the test? 8. Was the order of examination varied? 9. Was the time interval between repeated measurements compatible with the stability (or theoretical stability) of the variable being measured? 10. Was the test applied correctly and interpreted appropriately? 11. Were appropriate statistical measures of agreement used? %: final percentage of reliability (Items “yes” × 100)/11.
